# MicroRNAs in the prognosis of triple-negative breast cancer: A systematic review and meta-analysis

**DOI:** 10.1097/MD.0000000000007085

**Published:** 2017-06-02

**Authors:** Lingshuang Lü, Xuhua Mao, Peiyi Shi, Biyu He, Kun Xu, Simin Zhang, Jianming Wang

**Affiliations:** a Department of Epidemiology, School of Public Health, Nanjing Medical University, Nanjing; b Department of Clinical Laboratory, Yixing People's Hospital, Wuxi; c Department of Social Medicine and Health Education, School of Public Health, Nanjing Medical University; d The Innovation Center for Social Risk Governance in Health, Nanjing, China.

**Keywords:** biomarker, meta-analysis, microRNA, prognosis, triple-negative breast cancer

## Abstract

**Background:**

Triple-negative breast cancer (TNBC) is a heterogeneous group of tumors characterized by their aggressive nature and poor associated survival. MicroRNAs (miRs) have been found to play an important role in the occurrence and development of human cancers, but their role in the prognosis of TNBC patients remains unclear. We performed a meta-analysis to explore the prognostic value of miRs in TNBC.

**Methods:**

We systematically searched the PubMed, Embase, and Web of Science databases to identify eligible studies. A meta-analysis was performed to estimate the pooled hazard ratios (HRs) and their corresponding 95% confidence intervals (CIs) for the associations between levels of miR expression (predictive factors) and overall survival (OS) and disease-free survival (DFS) (outcomes) in patients with TNBC.

**Results:**

After performing the literature search and review, 21 relevant studies including 2510 subjects were identified. Six miRs (miR-155, miR-21, miR-27a/b, miR-374a/b, miR-210, and miR-454) were assessed in the meta-analysis. Decreased expression of miR-155 was associated with reduced OS (adjusted HR = 0.58, 95% CI: 0.34–0.99; crude HR = 0.67, 95% CI: 0.58–0.79). High miR-21 expression was also predictive of reduced OS (crude HR = 2.50, 95% CI: 1.56–4.01). We found that elevated levels of miR-27a/b, miR-210, and miR-454 expression were associated with shorter OS, while the levels of miR-454 and miR-374a/b expression were associated with DFS.

**Conclusions:**

Specific miRs could serve as potential prognostic biomarkers in TNBC. Due to the limited research available, the clinical application of these findings has yet to be verified.

## 1 Introduction

Breast cancer is the most commonly diagnosed malignancy and the leading cause of cancer-related mortality among women worldwide, with an estimated 1.7 million new cases and 521,900 deaths in 2012.^[1]^ Of the breast cancer cases, approximately 10% to 20% have been reported to be triple-negative breast cancer (TNBC).^[2]^ TNBC is a heterogeneous group of tumors characterized by the absence of estrogen receptor (ER), progesterone receptor (PR), and human epidermal growth factor receptor-2/Neu (HER2), and this malignancy has been found to be often, but not always, a basal-like breast cancer.^[3]^ Because it cannot be treated with current endocrine therapies and exhibits an aggressive nature, TNBC has been regarded as being associated with one of the worst prognoses of all breast cancer subtypes.^[2,4]^

Advances in in-depth research on genetic biomarkers, such as miRs, in TNBC have promoted the utility of biomarkers in the diagnosis, treatment, and prognosis of the disease. MiRs are a class of small noncoding RNA molecules that are 19 to 25 nucleotides in length, can modulate gene expression, and are easily accessible and quantifiable.^[5]^ A growing body of evidence indicates that aberrant expression of miRs may be linked with the development and progression of human cancers,^[6]^ including renal cell carcinoma,^[7]^ pancreatic ductal adenocarcinoma,^[8]^ and brain tumors.^[9]^ However, until now, no systematic review has been performed to explore the role of particular miRs in the survival of patients with TNBC.

In this study, we systematically reviewed relevant studies on the prognostic value of miRs in TNBC and pooled the effect estimates reported in these studies to provide a better understanding of associations between specific miRs and prognosis in TNBC and provide a rationale for miR-based therapeutics.

## 2 Materials and methods

### 2.1 Search strategy

We followed the guidelines of the Meta-analysis of Observational Studies in Epidemiology (MOOSE) group and Preferred Reporting Items for Systematic Reviews and Meta-analysis (PRISMA) statement.^[10]^ We searched the PubMed, Embase, and Web of Science databases to identify relevant studies that assessed the utility of candidate miRs as prognostic factors in TNBC. The databases were searched to identify articles published from January 1990 to December 2016 using the following search strategy: (microRNA OR miRNA OR miR) AND (triple-negative breast cancer) AND (prognosis OR prognostic OR survival OR outcome OR mortality). The searches were limited to articles published in English. Two investigators (LL and XM) reviewed the titles and abstracts of studies identified in the initial search to determine the relevance of these publications. Then, the full texts of the remaining articles were obtained and carefully reviewed. We also manually screened the reference lists of retrieved articles to identify other potentially relevant studies.

### 2.2 Eligibility criteria

Articles were considered eligible if they met all the following initial inclusion criteria: focused on patients undergoing treatment for TNBC; measured miR expression levels in tumor or blood samples; clearly defined the utilized miR cut-off points; clearly described the utilized miR detection methods; analyzed the correlations between survival outcomes and miR expression; and clearly described the follow-up duration. Articles were excluded if they were case reports, letters, commentaries, conference records or reviews; had a sample size less than 30 cases; calculated HRs based on a combination of multiple miRs; lacked sufficient data for estimating HRs and 95% CIs; or used survival data that originated from the TCGA, PROGmiR, METABRIC, or BreastMark dataset. Data were extracted from articles fulfilling all the aforementioned selection criteria. Two individual investigators (LL and XM) independently assessed the eligibility of the retrieved articles. Discrepancies were resolved by consensus or consultation with a third investigator (PS).

### 2.3 Quality assessment

The quality of the included studies was assessed according to the following checklist, which was developed based on the criteria proposed by the MOOSE group:^[10]^ clearly defined study design; clearly described study population (country); sufficiently large sample size (N > 30); clearly described the outcomes (OS or DFS); clearly defined the method of miR measurement (quantitative real-time polymerase chain reaction (qRT-PCR), in situ hybridization (ISH), etc.); clear defined the utilized cut-off values; measured miR expression in tumor or blood samples; and had a sufficiently long follow-up duration (>60 months). To assure the quality of this meta-analysis, studies were excluded if they did not meet these criteria.

### 2.4 Data extraction

Data were extracted independently by 2 investigators (LL and XM), who used a predefined sheet to retrieve information from all studies qualifying for final inclusion. The data sheet was designed based on previous studies focusing on similar topics and the PRISMA guidelines.^[11]^ The following data were extracted: title; first author; publication year; study design; study population; participant number; sample types; miRs; miR expression assessment methods; cut-off values; follow-up duration; and HRs for OS or DFS and their corresponding 95% CIs and *P* values. If HRs (95% CIs) and *P* values could not be extracted from the original article, we estimated these values using the available data or the Kaplan–Meier curves presented in the articles using the methods described by Parmar et al^[12]^ and Tierney et al.^[13]^

### 2.5 Statistical analysis

OS was defined as the interval from the date of primary surgery to the date of all-cause mortality. DFS was defined as the interval from the date of primary surgery to the date of disease relapse or all-cause mortality.^[14]^ We pooled the HRs (95% CIs) extracted from the studies using the Stata 13.0 software (StatCorp, College Station, TX). Heterogeneity was assessed using the Cochran Q test and Higgins *I*-squared statistic. *P* values less than .1 for the Q test and I^2^ value >40% indicated the presence of significant heterogeneity across studies. The fixed-effects model was applied in the absence of between-study heterogeneity, while the random-effects model was applied when heterogeneity was observed. An observed HR > 1 indicated worse prognosis in the group with elevated miR expression. Conversely, an observed HR < 1 indicated worse prognosis in the group with a decreased miR expression.^[15]^ Egger test was used to assess publication bias.

### 2.6 Ethical consideration

Ethical approval was not required for this study.

## 3 Results

### 3.1 Selection of studies

**Figure 1:**
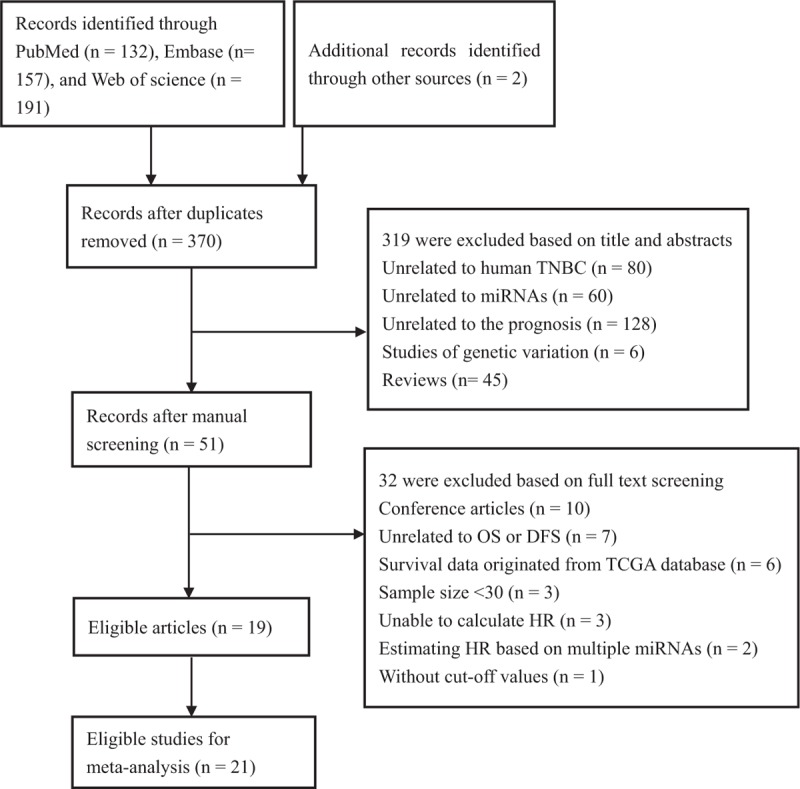
Flow diagram of the study selection procedure. DFS = disease-free survival, OS = overall survival, TCGA = The Cancer Genome Atlas.

A flow diagram of the study selection process is shown in Fig. 1. A total of 370 publications were identified in the initial search. After reviewing the titles and abstracts of these articles, we identified 51 articles evaluating the use of prognostic miR biomarkers in TNBC. We then carefully reviewed the full texts of these articles and excluded an additional 32 articles. Two articles described independent cohorts that were analyzed separately.^[16,17]^ Thus, we regarded these 2 articles as 4 studies. In total, 19 articles (21 studies) were eligible for inclusion in this meta-analysis.

### 3.2 Characteristics of the included studies

A total of 2510 TNBC patients were assessed in the 19 included articles, with a median sample size of 82 patients (range, 39–456 patients). These studies reported the prognostic values of 24 different miRs. The levels of miR expression were mainly detected in tumor tissues. Two studies used serum samples. Five studies did not directly report HR data. Thus, we estimated the HRs using the methods described above. Twelve studies reported adjusted HRs; the models described in these studies included covariates such as age, tumor site, grade, or disease stage (Table 1). In the included articles, increased expression of miR-27a/b,^[15,16,18,19]^ miR-34b,^[20]^ miR-210,^[14]^ miR-125b,^[21]^ miR-655,^[21]^ miR-21,^[22,23]^ miR-18b,^[24]^ miR-103,^[24]^ miR-107,^[24]^ miR-652,^[24]^ miR-301a,^[25]^ miR-30e,^[15]^ miR-214^[26]^ and miR-454^[17]^ decreased expression of miR-155,^[15,21,27,28]^ miR-16,^[21]^ miR-374a/b,^[19,21]^ miR-497,^[15,29]^ miR-493,^[15]^ miR-185,^[30]^ miR-26a,^[31]^ miR-126-3p,^[19]^ miR-218-5p^[19]^ and miR-361-5p^[32]^ were associated with poor prognosis in TNBC. Among these miRs, 6 (miR-155, miR-21, miR-27a/b, miR-374a/b, miR-210, and miR-454) were reported by at least 2 studies (Table 2). Thus, we performed this meta-analysis to summarize the effect of these 6 miRs.

**Table 1:**
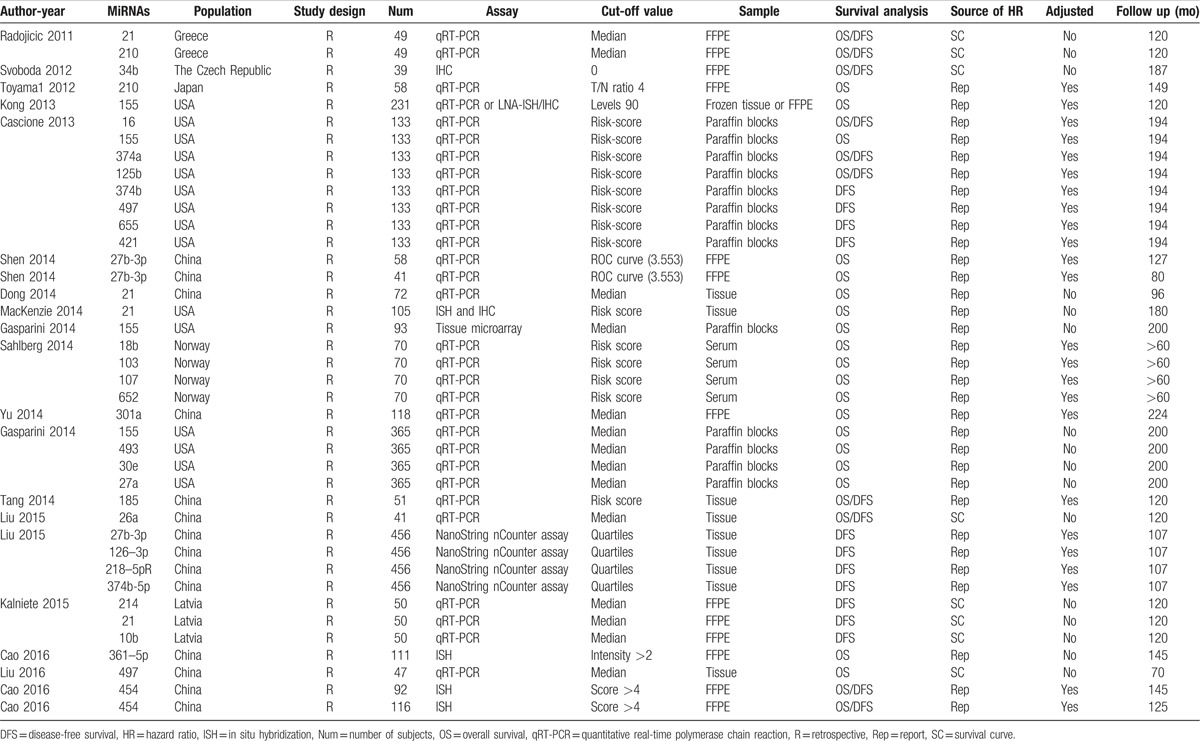
Main characteristics of the eligible studies.

**Table 2:**
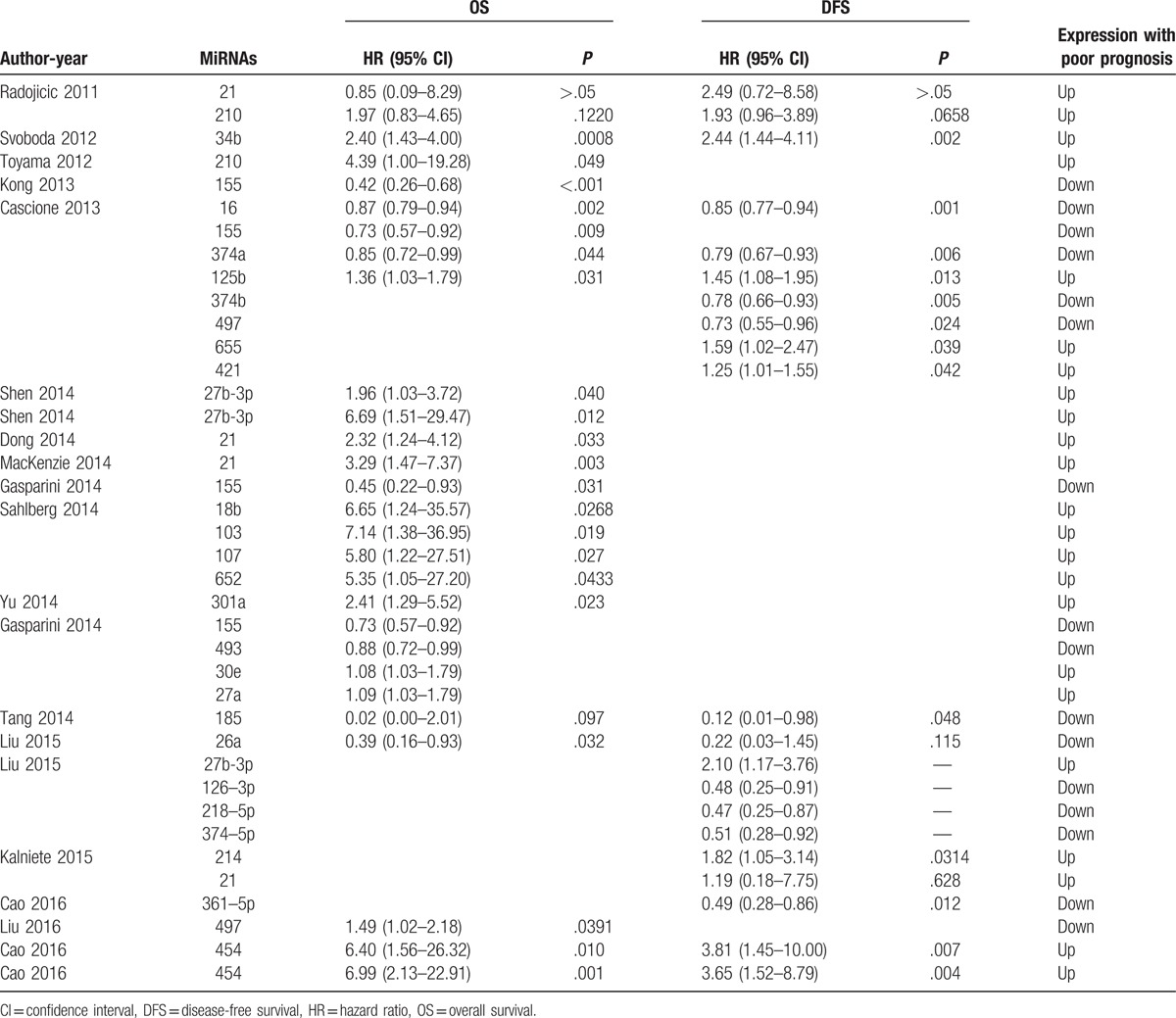
Descriptive characteristics and related data from included studies.

### 3.3 miR-155 and TNBC prognosis

**Figure 2:**
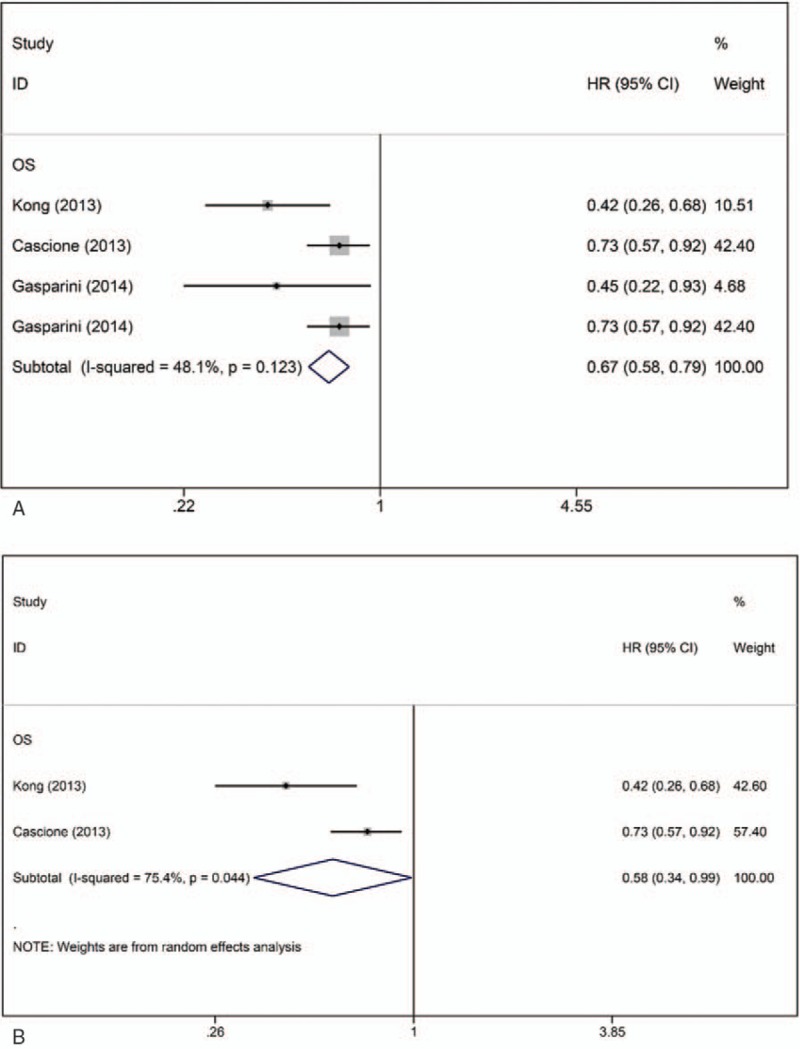
Forest plots of the HRs for the association between miR-155 and TNBC survival. A, Forest plot showing the combined HR based on all studies. B, Forest plot showing the combined HR based on multivariate studies. HR = hazard ratio, OS = overall survival.

Four articles (n = 822) suggested that downregulation of miR-155 was associated with poor prognosis in patients with TNBC. Gasparini et al^[15,27]^ calculated the crude HR for miR-155, while Kong et al^[28]^ and Cascione et al^[21]^ performed multivariate analyses. No significant interstudy heterogeneity was observed (I^2^ = 48.1%, *P* = .123), and the Egger test results indicated the presence of no significant publication bias (*P* = .091). The pooled crude HR was 0.67 (95% CI: 0.58–0.79) (Fig. 2A). If excluding studies only reporting crude HRs, the observed interstudy heterogeneity was significant (I^2^ = 78.4%, *P* = .044). The random-effects model revealed that miR-155 expression was consistently associated with OS in TNBC patients (HR: 0.58, 95% CI: 0.34–0.99) (Fig. 2B).

### 3.4 miR-21 and TNBC prognosis

**Figure 3:**
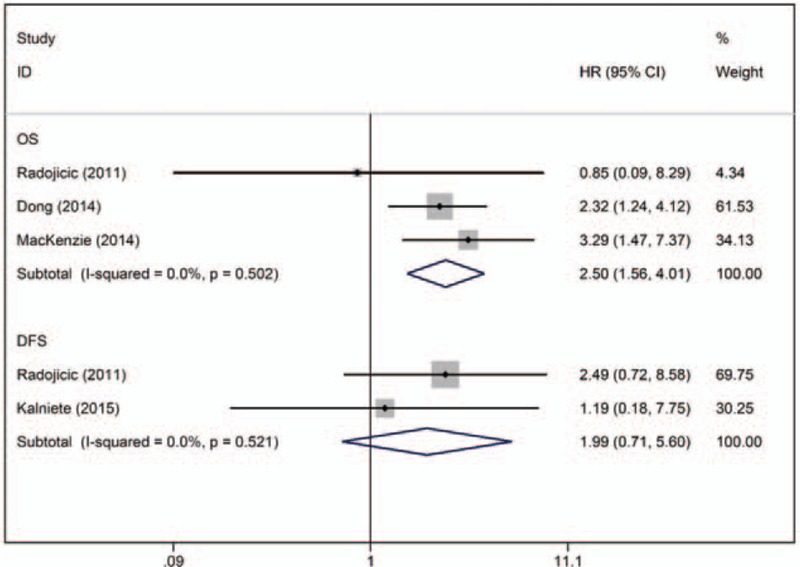
Forest plot of the HRs for the association between miR-21 and TNBC survival. DFS = disease-free survival, HR = hazard ratio, OS = overall survival.

Four articles (n = 276) reported the effect of miR-21 on the prognosis of TNBC patients. Of these studies, 1 reported both OS and DFS data,^[33]^ 2 reported only OS data,^[22,23]^ and 1 reported only DFS data.^[26]^ No significant heterogeneity was observed across studies (OS, I^2^ = 0.0%, *P* = .502; DFS, I^2^ = 0.0%, *P* = .521). The fixed-effects model revealed that miR-21 expression was inversely associated with OS (crude HR: 2.50; 95% CI: 1.56–4.01) and DFS (crude HR: 1.99; 95% CI: 0.71–5.60) in TNBC patients. There was no significant evidence of publication bias (OS, Egger test, *P* = .578) (Fig. 3).

### 3.5 miR-27a/b and TNBC prognosis

**Figure 4:**
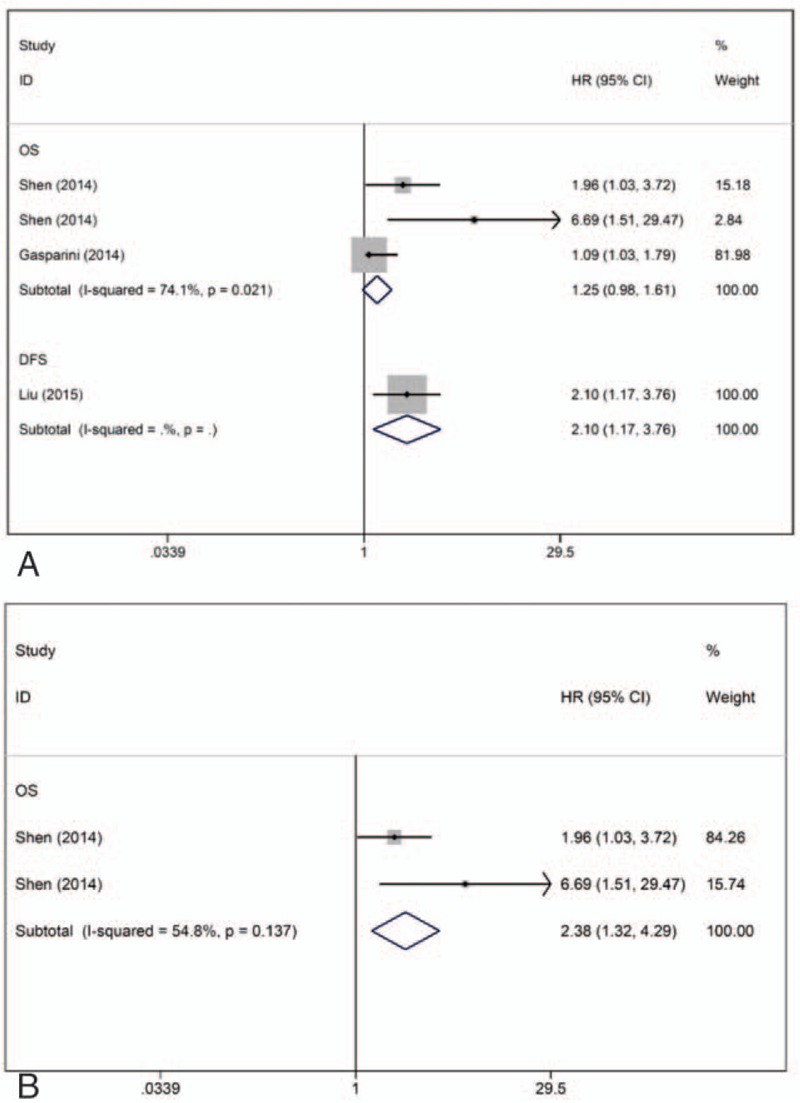
Forest plots of the HRs for the association between miR-27a/b and TNBC survival. A, Forest plot showing the combined HR based on all studies. B, Forest plot showing the combined HR based on multivariate studies. DFS = disease-free survival, HR = hazard ratio, OS = overall survival.

Three articles (4 studies, n = 920) assessed the association between miR-27a/b expression and prognosis in TNBC. Of these studies, 3 provided OS data,^[15,16]^ and 1 provided DFS data.^[19]^ For OS, a univariate HR was calculated in 1 study, while 2 studies performed multivariate analyses. The crude HR for the association between miR-27a/b expression and OS in TNBC was 1.25 (95% CI: 0.98–1.61) (Fig. 4A). After the univariate study conducted by Gasparini et al^[15]^ was excluded, the pooled adjusted HR for OS was 2.38 (95% CI: 1.32–4.29) (Fig. 4B).

### 3.6 The miR-374a/b and prognosis of TNBC

**Figure 5:**
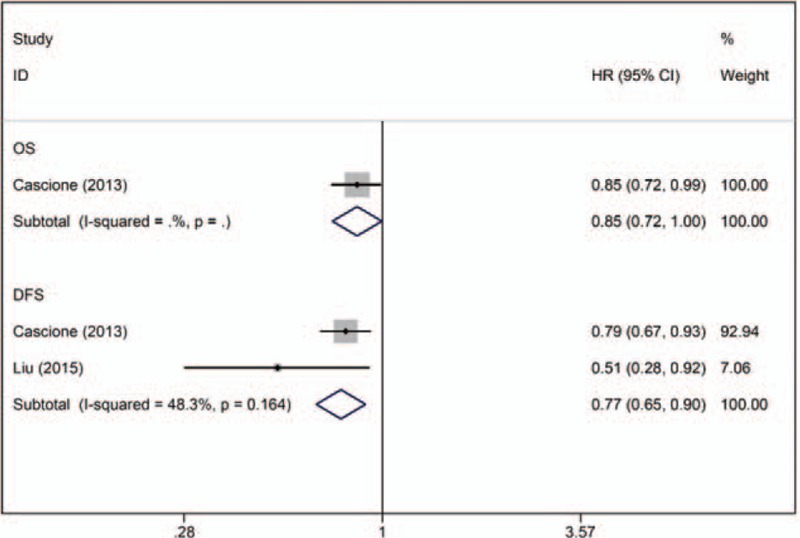
Forest plot of the HRs for the association between miR-374a/b and TNBC survival. DFS = disease-free survival, HR = hazard ratio, OS = overall survival.

Two studies evaluated the association between miR-374a/b expression and the prognosis of TNBC patients (n = 589), of which 1 reported data on DFS^[19]^ and 1 reported data on both OS and DFS.^[21]^ All of these studies provided adjusted HR data for DFS, and no significant heterogeneity was observed (I^2^ = 48.3%, *P* = .164). The fixed-effects model revealed that downregulation of miR-374 was associated with shorter DFS (combined adjusted HR: 0.77; 95% CI: 0.65–0.90) (Fig. 5).

### 3.7 miR-210 and TNBC prognosis

**Figure 6:**
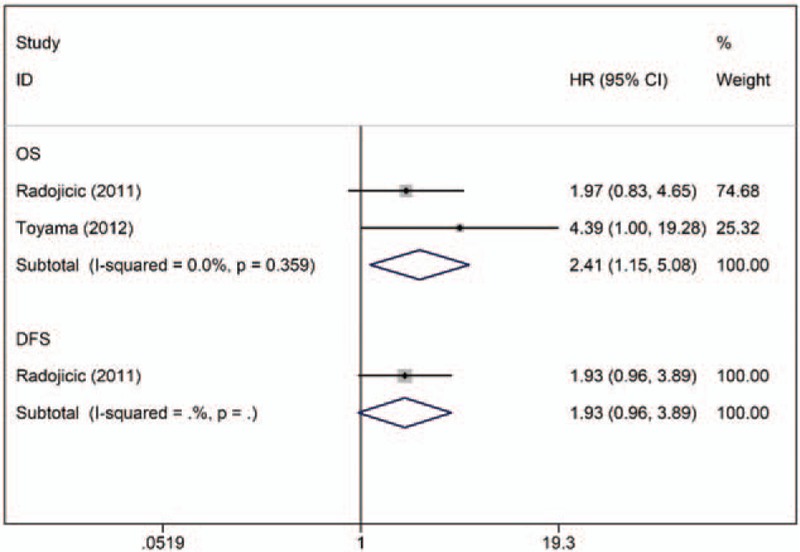
Forest plot of the HRs for the association between miR-210 and TNBC survival. DFS = disease-free survival, HR = hazard ratio, OS = overall survival.

Two studies determined the association between miR-210 expression and prognosis in TNBC (n = 107), of which 1 provided OS data^[14]^ and 1 provided OS and DFS data.^[33]^ For OS, no significant heterogeneity was observed across studies (I^2^ = 0.0%, *P* = .359). The fixed-effects model revealed that elevated miR-210 expression was predictive of shorter OS (crude HR: 2.41; 95% CI: 1.15–5.08) (Fig. 6).

### 3.8 miR-454 and TNBC prognosis

**Figure 7:**
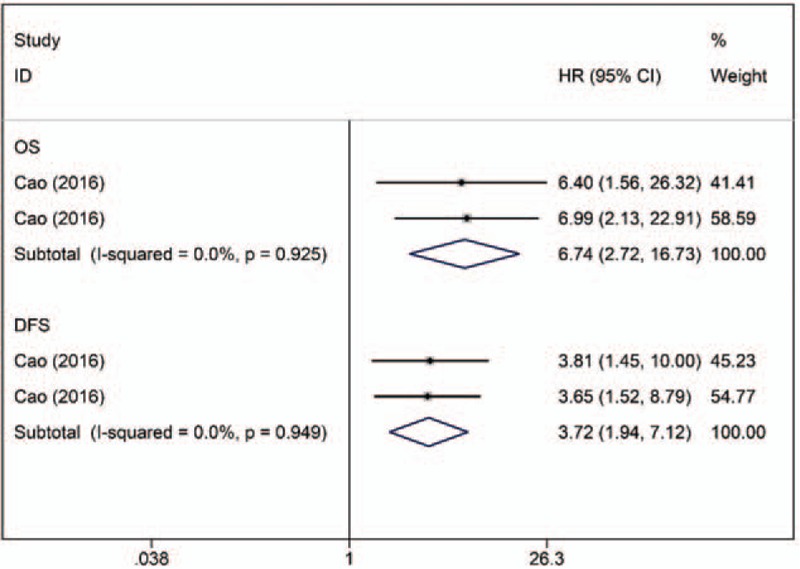
Forest plot of the HRs for the association between miR-454 and TNBC survival. DFS = disease-free survival, HR = hazard ratio, OS = overall survival.

One article describing 2 studies reported higher miR-454 expression to be a predictive factor for poor OS and DFS in TNBC using multivariate analyses (n = 208).^[17]^ No significant heterogeneity was observed across studies (OS, I^2^ = 0.00%, *P* = .925; DFS, I^2^ = 0.00%, *P* = .949). The fixed-effects model revealed that miR-454 expression was inversely associated with OS (combined adjusted HR: 6.74; 95% CI: 2.72–16.73) and DFS (combined adjusted HR: 3.72; 95% CI: 1.94–7.12) in TNBC patients (Fig. 7).

## 4 Discussion

We conducted a comprehensive systematic literature review to explore the utility of miR biomarkers that can be easily and robustly evaluated in predicting prognosis in TNBC. To our knowledge, this is the first extensive meta-analysis to describe the role of miRs in TNBC prognosis.

Although various miRs were found to be associated with prognosis in TNBC, most of these miRs were assessed in only a single study. Six miRs (miR-155, miR-21, miR-27a/b, miR-374a/b, miR-210, and miR-454) were evaluated in at least 2 studies. We, therefore, performed a meta-analysis of the effect of these 6 miRs on the survival of TNBC patients. The results of this study showed that lower expression of miR-155 predicted worse OS in TNBC patients, while elevated levels of miR-21, miR-27a/b, miR-210, and miR-454 expression were associated with shorter overall survival times. Similarly, lower expression of miR-374a/b and higher expression of miR-454 were associated with shorter DFS.

The miR-155 locus is located within a region known as B-cell integration cluster,^[34]^ and miR-155 is overexpressed in various solid tumors, including breast, lung, colon, pancreatic and thyroid cancers.^[35,36]^ Some studies have reported the pro-oncogenic properties of miR-155 in lung cancer^[36]^ and T-cell leukemia.^[37]^ However, we identified this miR to exhibit opposite behavior, finding that overexpression of miR-155 tended to have a protective effect on survival in TNBC patients. There are a number of molecular mechanisms that could explain this relationship. In TNBC, miR-155 may play a crucial role in DNA damage pathways.^[15]^ miR-155 may regulate DNA repair activity and sensitivity to ionizing radiation by repressing RAD51 recombinase (RAD51),^[27]^ while RAD51 has been identified as a central protein in homologous recombination.

MiR-21 is one of the most extensively studied cancer-related miRs and might play an ever-expanding role in most cancers.^[38]^ miR-21 may serve as a key regulator of oncogenic processes, including tumor growth, migration, and invasion.^[39]^ Elevated miR-21 expression levels have been found to be associated with poor outcomes in cancer patients.^[40]^ miR-21 may target the pro-apoptotic phosphatase and tensin homolog (PTEN) and promote tumor cell proliferation, which, in turn, may inhibit the apoptosis of tumor cells in TNBC cell lines in vitro.^[22]^

miR-27a/b has been linked to the peroxisome proliferator-activated receptor (PPAR) and PTEN signaling in TNBC cells, acting as a tumor suppressor by regulating the cell division cycle (CDC27) gene.^[19]^ CDC27 has been identified as a core component of the anaphase-promoting complex (APC) and found to be involved in regulating mitotic checkpoints to ensure chromosomal integrity.^[41]^ The results of a pathway analysis showed that miR-374b may regulate critical pathways involved in TNBC tumor development and progression, including the fibroblast growth factor and transforming growth factor pathways.^[19]^ miR-210, a known hypoxia-regulated miR, has been found to be upregulated in many cancers. This miR may serve as a key player in cell response to hypoxia and has been linked to a number of hypoxia-dependent diseases involved in mitochondrial metabolism, angiogenesis, DNA repair, and cell survival.^[42]^ miR-454 has dual functionality, acting as either an oncogenic miR or a tumor suppressor. Previous studies have reported this miR to be downregulated in esophageal cancer^[43]^ and upregulated in colorectal cancer and breast cancer.^[44,45]^ miR-454 has been reported to function as an oncogenic miR by targeting PTEN. Patients with TNBC tumors that lose PTEN expression have poorer survival, as PTEN negatively regulates the PI3K-AKT signaling pathway.^[46,47]^ Further studies are needed to understand the molecular mechanism underlying the effect of miRs in TNBC.

Some limitations must be considered when interpreting the results of the current study. First, the number of studies available was limited. More studies are needed to further assess these associations in the future. Second, marked heterogeneity was observed in some of the analyses, findings that were likely identified due to differences in patient characteristics (ethnicity, nationality, gender, age, tumor stage, and grade) and the use of different assay methods, cut-off values for miR expression levels, sample preparation methods (i.e., paraffin-fixed, formalin-fixed, freshly frozen tumors or serum), follow-up durations, and HR extraction methods. Third, circulating biomarkers are more valuable than tissue biomarkers because they can be assayed before surgery and monitored throughout the lifespan. More studies should be conducted in the future to evaluate the prognostic value of specific miRs in serum in TNBC.

## 5 Conclusions

Specific miRs may serve as potential prognostic biomarkers in TNBC. Due to the limited research available, the clinical application of these findings has yet to be verified.
